# Lessons from across the pond: Student perspectives on the Internal Medicine clerkship experience at an Irish and Canadian medical school

**DOI:** 10.15694/mep.2020.000016.1

**Published:** 2020-01-15

**Authors:** Ryan Taylor Sless, Nathaniel Edward Hayward, Paul MacDaragh Ryan, Adam Kovacs-Litman, Umberin Najeeb

**Affiliations:** 1 University College Cork; 2 University of Toronto

**Keywords:** Medical Education, International Medical Graduates, Clerkship, Canada, Ireland, Internal Medicine

## Abstract

This article was migrated. The article was marked as recommended.

There is an increasing number of Canadians studying medicine outside of Canada, with a large cohort studying in Ireland. Studying abroad often means different foci in medical training which may make transitioning to residency in a different system more challenging. Students often enter North American elective rotations with little knowledge of student roles and responsibilities. This paper provides insight into the differences in learning objectives and student experiences in an Internal Medicine clerkship at a medical school in Canada and Ireland. Learning objectives are similar between systems; but there is an experiential discordance. In Ireland, clerks see many different patients, gaining exposure to a breadth of topics and clinical signs, but medical student presentations rarely inform decisions around patient care. In Canada, clerks have more direct patient responsibilities, performing physical examinations, reviewing investigations, writing progress notes, and devising management plans as part of their professional development. Overall, the Irish system places emphasis on the mastery of core clinical skills and maximizing breadth of patient exposure whereas the Canadian clerkship is more focused on graduated responsibility and formulating management plans, at the expense of some breadth of exposure. Such discrepancies may not affect the quality of residents, but are important considerations for Canadians studying abroad when repatriating for electives and residencies.

## Introduction

As residency beckons, international medical students intent on returning to Canada question how their training compares to Canadian-trained students. There are over 3500 Canadians studying medicine abroad at 80 different medical schools in 30 different countries, with the majority studying in Australia and Europe (within which Ireland hosts the largest portion) ( CaRMS, 2010; Barer *et al.*, 2014). Upon completion of core rotations in Ireland, several of the authors completed clerkship rotations in Canada. We didn’t know what to expect. Irish-trained students commonly rely on vague informal commentary from far-removed ‘doctor friends’ and urban legends of repatriated alumni. North American electives are critically important for residency matching. Students are evaluated on their performance and relationships made in this short time are integral to securing a residency position. A *post hoc* comparison has limited utility for students aiming to prove their readiness upon arrival at these early career-defining rotations. In this paper, we outline a comparison of Internal Medicine Learning Objectives of an Irish medical school (University College Cork) and CanMEDs Clerkship Curriculum Objectives for a Canadian medical school (University of Toronto) for the specialty of Internal Medicine ( Table 1). We subsequently provide a subjective experiential comparison by way of personal observation. We hope this narrative may interest our student successors in Ireland and International Medical Graduates (IMGs) hoping to pursue residency in Canada.

## Comparison of Learning Objectives

Upon review of Learning Objectives, major differences were not identified in curricula for pathophysiology, clinical medicine, and physical examination skills ( Table 1) ( University College Cork School of Medicine, 2018; University of Toronto Faculty of Medicine, 2018). The majority of identifiable differences were in areas relating to law, epidemiology, and public health. Irish trainees do not receive training on Canadian health policy or risk factors unique to Canadian populations. Some examples include: differences in responsibility for end-of-life decision-making and code status (these decisions are made by the patient or substitute decision maker in Canada, but by the most responsible physician in Ireland) (Medical Council of Ireland, 2017), epidemiological differences in disease prevalence (e.g. high rates of celiac disease and cystic fibrosis in Ireland) ( Johnston *et al.*, 1997; Farrell, 2008), and other socio-medical issues such as healthcare access for specific populations including the First Nations, Inuit, and Métis peoples in Canada and Travelling Community in Ireland ( Harfield *et al.*, 2015; O’Donnell *et al.*, 2016).

**Table 1.** Direct comparison of the Internal Medicine Learning Objectives from University College Cork (Cork, Ireland) with the Internal Medicine Learning Outcomes from the University of Toronto (Toronto, Canada), grouped by CanMEDs roles of a medical student.

Quality	Ireland - UCC Internal Medicine Learning Objectives	Canada - University of Toronto Internal Medicine Clerkship Curriculum Objectives
**Medical Expert**	Demonstrate understanding of basic medical sciences and knowledge of the core topics in Medicine and Surgery	Demonstrate knowledge of the scientific and humanistic foundations of medicine, as learned during the pre-clerkship and expanded on during the clerkship, in order to more rationally diagnose and manage the various factors contributing to a patient’s illness.
	Demonstrate competence in presenting a complete history and performing a physical examination on an adult patient.	Students should be able to obtain and document both a complete and a focused medical history, as the situation requires. The history will be thorough and organized, and supplemented as needed by information from other sources (family members, other health care institutions medical records, other physicians, etc.). Students should be able to perform and document both a complete and a focused physical examination, as the situation requires.
	Interpret clinical data and discuss the diagnosis and management of common acute and chronic conditions in adults	Students should be able to interpret commonly-employed diagnostic tests. Students should be able to integrate the above history, physical findings and diagnostic test results into a meaningful diagnostic formulation. Students should be able to demonstrate therapeutic and management skills.
**Communicator**	Demonstrate effective communication skills in all areas including interaction with patients, families and their environment.	Communicate effectively with patients, their families and the community through verbal, written, and other non-verbal means of communication.
	Demonstrate competence in presenting a complete history	Present a case summary orally in a clear, logical, and focused manner.
**Collaborator**	Adopt a multidisciplinary approach to clinical practice.	Participate in interdisciplinary team discussions, demonstrating the ability to accept, consider, and respect the opinions of other team members, while contributing an appropriate level of expertise to patient care.
**Manager/Leader**	Exhibit professional behaviour by meeting all scholarly requirements in a professional and timely manner	During the clerkship in internal medicine, the medical student will deepen his/her understanding of the appropriate use of health care resources in the internal medicine context and contribute to a culture that promotes patient safety. Students are also expected to manage their own time in an efficient manner.
**Health Advocate**	Demonstrate competence in health promotion and disease prevention in adults	Recognize important determinants of health and principles of disease prevention pertinent to internal medicine.
		Act as an advocate on behalf of patients assigned to their care, when interacting with other members of the health care team.
**Scholar**	*Self-directed learning is a significant part of the curriculum, with a list of learning objectives that students must cover however there is not a specific learning objective in the curriculum outline to this topic.*	Demonstrate the ability to engage in self-directed learning. This involves identifying personal learning objectives, and then finding and using a variety of resources to address learning needs, and to use self-reflection to assist their own learning.
**Professional**	Adopt an empathic, holistic approach to patients and their problems.	Demonstrates sensitivity to patients’ and others’ needs, including taking time to comfort the sick patient. Listens with empathy to others
	Exhibit professional behaviour by meeting all scholarly requirements in a professional and timely manner	Fulfills obligations in a timely manner, including transfer of responsibility for patient care
	Demonstrate effective personal, professional and advanced consultation skills	

## Experiential Comparison

### Clerkship in Ireland

A clerkship day in Ireland typically includes: 1-2 group-based tutorials, 1 hour of bedside teaching, a team ward round, and histories/physical examinations on 2-3 patients. Patient interactions are commonly limited to patients familiar to the attending physicians or junior doctors. Clerks are expected to present structured histories and examination findings, but these presentations rarely inform decisions around patient care. Constructive criticism and questions about the pathophysiology and management of relevant conditions commonly follow. Each day, clerks see different patients for practice, learning about a breadth of topics and seeing many clinical signs. There is a strong emphasis on bedside teaching where a consultant takes a group of students to patients with known diagnoses. Irish clerks routinely perform basic procedural skills such as phlebotomy and intravenous cannulation, as this is a fundamental function of the junior doctor within the Irish healthcare system. Overnight call is not a requirement, though students can elect to be on-call with their assigned teams.

### Clerkship in Canada

In Canada, the clerkship format is different in many ways. Observations are primarily drawn from Clinical Teaching Units (CTUs) and select Internal Medicine subspecialty rotations. A clerkship day in Canada typically includes: 1-2 group-based tutorials, 1-2 hours of structured bedside teaching per week, reviewing active patient issues with the team, direct responsibility for 2-6 patients (numbers vary based on patient volumes and level of training), seeing new undifferentiated patient consultations, and overnight call. Clerks are responsible for following up active patient issues, performing physical exams, reviewing investigations, writing progress notes, and creating management plans, which are reviewed by the team. This format challenges clerks to apply clinical skills, follow the same patients each day, observe the effects of their management, and prompt adjustments as necessary. Overnight call is an expectation of Canadian clerkship where medical students see new consultations from the emergency department and manage overnight issues for their admitted ward patients, overseen by a senior medical resident (PGY2 or PGY3).

## Discussion

While the Medical Councils of both Canada and Ireland have similar expectations for the core competencies of their students and trainees, there are unique aspects of each program. In Ireland, there is a greater focus on the mastery of core skills (including basic procedures) and maximizing breadth of exposure by seeing different patients every day, at the expense of direct patient responsibility. Canadian clerkship is more focused on graduated responsibility through individual patient interactions, formulating management plans, and a greater role on a healthcare team, at the expense of some breadth of exposure.

As globalization continues to universalize the supply of medical graduates, greater emphasis should be placed on understanding transatlantic differences in clerkship curriculums both in terms of education and patient-care responsibilities. Publicizing these differences may also better allow preceptors to evaluate students in the context of their training. Awareness of these differences may allow students and preceptors to better align expectation frameworks, making for an even more positive elective experience for all.

There are many possible explanations for differences in Irish and Canadian clerkship experiences ranging from cultural to practical. The Irish and Canadian medical systems evolved in different ways and developed different medical traditions including the delegation of patient care responsibilities. Canada abolished the rotating internship in the early 1990s whereas the Internship year remains a mandatory component of medical training in Ireland. There is no rush for medical students to assume patient care responsibilities in Ireland as Internship serves as a foundational year, allowing learners to achieve graduated responsibility in patient care. Irish residency programs are also on average longer than their Canadian counterparts. Differences in patient volumes may also account for some differences, but no direct comparisons exist in the literature. Noted differences are unlikely to be specific to Internal Medicine. A more thorough comparison amongst a broader range of specialties is warranted to outline roles and responsibilities for incoming international elective students.

## Take Home Messages

• There is an increasing number of Canadian’s travelling abroad to complete their medical education.
• The learning objectives for clerkship rotations in Canada and Ireland are very similar.
• Clerkship in Canada focuses on graduated responsibility and patient management.
• Clerkship in Ireland focuses on mastery of core skills and breadth of exposure.

## Notes On Contributors

Ryan Taylor Sless - Ryan Sless is a final year medical student at University College Cork in Cork, Ireland who plans to pursue a career in Internal Medicine. In addition, he has a Master’s of Science in Cardiovascular Exercise Physiology from the University of Toronto and a special interest in Simulation and Medical Education. ORCID ID: https://orcid.org/0000-0001-5086-426X

Nathaniel Edward Hayward - Nathaniel is a Canadian medical student currently studying at University College Cork School of Medicine in Cork, Ireland. He is completing his final year of study and plans to pursue a residency in pediatrics. In his spare time, he is an avid distance runner, coffee connoisseur, and music lover. ORCID ID: https://orcid.org/0000-0001-9085-981X

Paul MacDaragh Ryan - Paul MacDaragh Ryan is a final year medical student at University College Cork and an Evidence Synthesis Ireland Fellow within the Grimshaw group in University of Ottawa. Paul holds a PhD in gut microbiome research and continues projects in the field within the Centre for Research in Vascular Biology. ORCID ID: https://orcid.org/0000-0001-7251-6725

Adam Kovacs-Litman - Adam Kovacs-Litman is an Internal Medicine resident at the University of Toronto. He obtained his medical degree from Western University and undergraduate from Queen’s University. He has an interest in Quality Improvement with ongoing research using covert and electronic monitoring to quantity hand hygiene rates and implications for patient care.

Umberin Najeeb - Dr. Najeeb is staff internist at Sunnybrook Health Sciences Centre and an Assistant Professor of Medicine at University of Toronto. She is the Faculty-Lead for International Medical Graduates [IMGs] mentorship program and the Co-Director of Department of Medicine’s Master Teachers Program at UofT. ORCID ID: https://orcid.org/0000-0002-1652-9959
