# Supplementary material for: Lessons from across the pond: Student perspectives on the Internal Medicine clerkship experience at an Irish and Canadian medical school
Source: MedEdPublish (2016). 2020 Jan 15;9:16. [Version 1] doi: 10.15694/mep.2020.000016.1 (PMC10702639; doi:10.15694/mep.2020.000016.1)
Supplement: Peer review history

# Reviewer report: Reviewer response for version 1

**DOI:** 10.21956/mep.19965.r30343

**Published:** 2020-05-12

**Authors:** Ken Masters

**Affiliations:** 1 Sultan Qaboos University

This review has been migrated. The reviewer awarded 4 stars out of 5 And interesting opinion piece paper giving student perspectives on the Internal Medicine clerkship experience at an Irish and Canadian medical school.Although the focus of the paper is on the student experience, one of its valuable contributions is the description (including Table 1), of the differences and similarities in the LOs of the two systems, and how strongly (of necessity) they are informed by local concerns and conditions.The description of various processes, although a little thin on detail, also adds interesting perspectives. For value to future Canadian medical students going to Ireland (and vice versa), this is a useful opinion piece, and would be good reading. In addition, curriculum designers might find the similarities and differences interesting.I would like to see the authors develop this opinion piece into a full-blown (probably qualitative) study, so that more in-depth issues could be highlighted. Still, as an opinion piece, this is a valuable read.

Reviewer Expertise:

NA

No decision status is available

# Reviewer report: Reviewer response for version 1

**DOI:** 10.21956/mep.19965.r30342

**Published:** 2020-03-05

**Authors:** Trevor Gibbs

**Affiliations:** 1 AMEE

This review has been migrated. The reviewer awarded 3 stars out of 5 An interesting paper written to demonstrate that what we often take for granted is not always what happens in real-time. We frequently assume that equity exists between two curricula , especially from schools of similar disposition and resource. The frequent travel between countries and schools that we now see among both undergraduate and postgraduate students causes curriculum planners and programme developers to assume too much, as this paper proposes.The paper was a little short on how this dichotomy of outcomes could be resolved and whether the outcomes were standard or reflected the needs of the country- this I think would make it a more rounded paper that was useful to all readers.. I would still however recommend the paper to curriculum planners for their discourse on the subject.

Reviewer Expertise:

NA

No decision status is available
